# Climate and human-modified landscapes influence spread of invasive agricultural pest *Popillia japonica* Newman in American Midwest and Great Plains

**DOI:** 10.1038/s41598-025-18493-x

**Published:** 2025-09-26

**Authors:** Nicole B. Kucherov, Trevor Hefley, Tania N. Kim

**Affiliations:** 1 Department of Entomology, Kansas State University, 123 W. Waters Hall 1603 Old Claflin Place, Manhattan, KS 66506 USA; 2 Department of Statistics, Kansas State University, 101 Dickens Hall 1116 Mid-Campus Drive N, Manhattan, KS 66506 USA

**Keywords:** Agriculture, Climate impact, Developed areas, Grassland, Invasive pest, Invasion dynamics, Agroecology, Invasive species

## Abstract

Japanese beetle (*Popillia japonica)* is an invasive agricultural pest throughout the eastern United States and its distribution is expanding westward. Understanding how climate and land use intensification interact to affect Japanese beetle population dynamics and spread is essential to predict which areas are vulnerable to increased pest pressure. In this study, we combine data from the Global Biodiversity Information Facility (GBIF) with data from local state agricultural agencies and University extension offices to model how land cover and climate affect Japanese beetle occurrences at the leading edge of an invasion front in the central United States over 5 years. We found that beetle occurrences were positively correlated with proportion of developed areas, proportion of corn and soybean, minimum temperature, and precipitation within counties, and negatively correlated with the proportion of grassland and pasture, and maximum temperature. We modeled Japanese beetle occurrences in the near future and for future climate scenarios and found high probability of occurrence for Japanese beetles across the entire region. Our study highlights the importance of supplementing community science data in rural areas and including a time variable when assessing habitat associations for invasive species in novel areas.

**Supplementary Information:**

The online version contains supplementary material available at 10.1038/s41598-025-18493-x.

## Introduction

Changing environments, caused by climate and land cover change, are negatively affecting pest dynamics in agroecosystems and threatening food security. To meet growing demands for food, global agricultural production must double by 2050, and the suggested way is to intensify crop yield production rather than expand agricultural land^1^. However, agricultural intensification has led to increased pesticide usage with negative effects on non-target species such as wildlife and beneficial insects^2^, harm to human health^3^, and increased insecticide resistance^4,5^. These outcomes are exacerbated with changes in temperature and weather patterns which will affect insect life cycles, survivability, and pest outbreaks^6–9^ as well as relationships between pests and their natural enemies^2,10,11^. Invasive species are particularly problematic, causing a reduction in food supply with global costs of $279 billion USD in 2019^12^, and their impact is expected to rise with increased global trade^13^ and climate change^9^. Therefore, understanding how climate and land use intensification interact to affect invasive pest population dynamics and spread is essential for effective and targeted management efforts.

One invasive pest that is of major concern in the United States is the Japanese beetle (*Popillia japonica* Newman*)*. Japanese beetles feed on over 300 plant species, including turfgrass, horticultural plants, fruit crops, soybean, corn, and other field crops^14–16^. Native to Japan, Japanese beetles were introduced to the United States in 1916 through a nursery in New Jersey^17,18^ and by 1998, they quickly spread to all states east of the Mississippi river, except Florida^19^. As of 2015, there were established populations in 28 eastern states and moderate populations in states west of the Mississippi River^19^. Japanese beetles have recently become a growing concern for the Midwestern United States, due to their potential to damage corn and soybean production^16^. Japanese beetles defoliate soybean plants, causing severe damage if they defoliate the leaves during a reproductive stage (e.g., approximately 20% yield loss after 30% defoliation^20^). In corn, Japanese beetles clip the silks, which interferes with pollination, causing a reduction of kernels^21^. The economic impact of Japanese beetles in the United States was estimated as $450 million per year by ARS in 2010^22^. However, it is likely to be far higher now. A recent report estimated economic damage by the Japanese beetle in the state of Utah would be $126 million in 2027^23^. Even with current controls and quarantines, Japanese beetle expansion continues to threaten agriculture and horticulture as they move westward in the United States.

Investigating how invasive species, such as the Japanese beetle, expand their distribution is challenging for many reasons^24^. Current models studying the expansion of Japanese beetle rely on occurrence data from different regions to determine habitat associations within a novel region which can be problematic especially along invasion fronts such as the Midwestern US^19,25,26^. For example, Zhu et al.^27^ found that habitat association models using Japanese beetle occurrence, climate and land cover data from the native range in Japan was not able to explain Japanese beetle distribution within the introduced range in North America. This discrepancy could arise because invasive species within a novel habitat have not filled their ecological niche, and therefore absences could simply be due to not having enough time to reach unoccupied areas rather than negative associations^28^. As such, habitat associations within the native range (where all ecological niches have been occupied) can be different from the associations within the non-native range^29^. Additionally, the spatial extent can affect habitat associations as climate factors may better correlate with species occurrences at a global scale due to greater variability in climate at larger spatial scales, while environmental factors may better explain the occurrence of species at finer scales due to greater variability at these smaller spatial scales^30^. Therefore, to improve our understanding of how Japanese beetles utilize landscapes and predict their regional occurrences in the future, we need to create habitat association models at the appropriate spatial extent and location by incorporating regional environmental, climatic, and anthropogenic factors.

Another challenge for understanding habitat associations is the quality of the occurrence data used to create the statistical model. Invasive species present unique spatial biases because eradication efforts can obscure natural habitat associations, sampling protocols can differ across political boundaries, and sampling efforts may vary in the long-established versus the recently invaded areas^31^. Additionally, studies that pool all occurrence observations together as one point in time and then predict the distribution for a future time or climate scenarios^19,25,26^ can be problematic because this approach assumes that the population of the modelled species is currently in equilibrium with its environment^28,32^. When modeling invasive species, habitat associations can be obscured simply because the invasive species has not yet spread to a given location^33^. Sampling bias is also introduced when using community science data which allows data to be collected across large geographical areas and over a long period of time^34^. These sampling biases include missing data which might confound data gaps in unsampled areas from true absences^35^ and variation in observation effort with more observations occurring in populated areas and incomplete detection in less populated areas^36^. Due to the nature of community science, observations are collected in areas where users spend their time, while large expanses of private property are not explored, leading to the underrepresentation of agricultural and rural areas. Therefore, biased occurrence data could be misleading in understanding habitat associations, and an effort must be made to incorporate data from other sources.

For this study, we examine habitat associations of Japanese beetles, *Popillia japonica*, and predict their occurrences along the invasion front within the Great Plains and Midwest regions of the United States. We address the challenges outlined above to model habitat associations of Japanese beetles in the states of Iowa, Kansas, Missouri, and Nebraska, United States, over a five-year period (2017–2021). By using data from this time and spatial range, we can capture how Japanese beetles are utilizing landscapes at the leading edge of an invasion front and predict future spread. We use community science occurrence data from the Global Biodiversity Information Facility, GBIF (www.gbif.org), along with presence-absence data from state agricultural agencies and University extension offices to overcome spatial biases associated with public databases. This study is the first to combine two such datasets to analyze the effects of land cover and climate variables on the spread of the Japanese beetle in a novel area. The addition of observation data from state agricultural agencies and extension offices is integral to understanding how this invasive agricultural pest spreads in an agriculturally dominated landscape such as the American Midwest. We hypothesized that Japanese beetle presence will have a positive relationship with developed areas, as well as grasslands and pasture, and agricultural land due to availability of food resources. We also hypothesized a positive relationship between Japanese beetle presence and precipitation, as these beetles prefer warmer temperatures and moist soil^14,15^. We hypothesized that lower precipitation would result in lower probabilities for Japanese beetle presence in the western portion of this region, especially under possible future drier climatic conditions. We then used our habitat association model to understand and predict how Japanese beetles are spreading into novel areas of this region. This knowledge could be used to guide mitigation efforts of the beetle and minimize its impact on important agricultural production in the central United States.

## Methods

### Study area

**Fig. 1:**
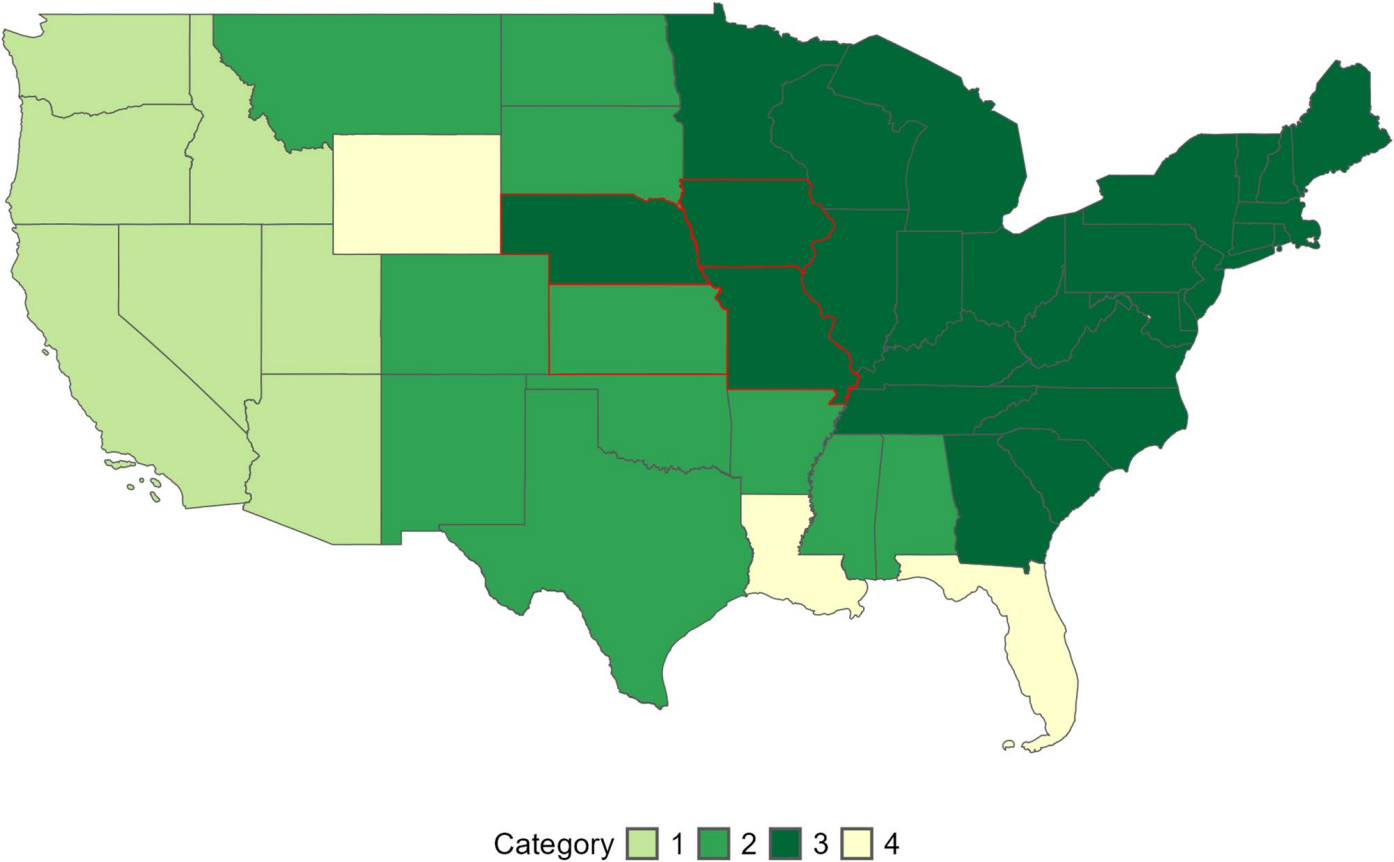
United States map of Japanese beetle (Jb) infestation status by state. States listed as “Category 1” are uninfested and Jb are considered quarantined pests. “Category 2” are uninfested or partially infested states and Jb are regulated but not quarantined. “Category 3” are partially or generally infested state, and Jb are not regulated. “Category 4” states are not known to be infested. (modified from *Japanese Beetle Harmonization Plan*, 2022)

For this study, the four states of Iowa, Kansas, Missouri, and Nebraska were chosen due to their location along the western invasion front of the Japanese beetle in the United States (Fig. 1). Assuming that an invasive species will have the same habitat associations across its entire range can lead to false predictions^29^therefore here we focus on habitat associations within this region to better infer relationships between the spread of Japanese beetles and regional landscape and climate factors. According to recent models, the climate-suitable range of Japanese beetles within the United States remains partially unfilled, and some un-infested areas are at risk for invasion now, under current climate conditions^27^. Additionally, there is concern that the managed pastures of this region will provide ideal habitat for Japanese beetle larvae^19^. In the four years between 2018 and 2022, Nebraska’s infestation status changed from partially infested to generally infested, indicating an advancing western invasion into the Great Plains (Fig. 1).

### Data collection

We obtained occurrence data on Japanese beetle observations from Global Biodiversity Information Facility, GBIF^37^. Data were downloaded using a structured search for all entries of the species “Popillia japonica Newman, 1838” entered as a human observation within the United States and including coordinates. This search led to 16,493 observations^37^. Observations which did not include data on “State/province” were omitted. We filtered the GBIF observations down to the four states (Iowa, Nebraska, Missouri, and Kansas) and years (2017–2021) of our study, resulting in 1141 observations. There were only 39 observations within these states prior to 2017.

We gathered our supplementary data from state agricultural sources through presence/absence county maps provided online and personal correspondence. The state data from Iowa was compiled through reports of Japanese beetle sightings from landowners and extension officials and retrieved through personal correspondence (D. Lewis, personal communication, 29 Jan 2024). In Nebraska and Kansas, the state’s Department of Agriculture is actively monitoring and trapping Japanese beetles. For Nebraska, we used the presence/absence county maps provided online by the Department of Agriculture^38^. For Kansas, we obtained trapping data from the Department of Agriculture (R. Wilkins, personal communication, 29 Apr 2024). For Missouri, we received trapping data from a monitoring effort based at University of Missouri (K. Rice, personal communication, 9 Nov 2022).

Land cover data were downloaded by county from USDA’s Cropland data layer (CDL)^39^ using the CropscapeR package^40^. For each county, we calculated the percentage of land cover that was designated as corn, soybean, grassland and pasture, and developed areas. The developed land cover variable is the sum of the low, medium, and high intensity, as well as developed open space areas because they all represent human-dominated landscapes where lawns and turf would be present. County level climate variables for average annual temperature, annual maximum temperature, annual minimum temperature, and total annual precipitation between 2017 and 2021 were downloaded from NOAA’s Climate at a Glance County Time Series^41^.

### Data conversion

To help counter the community science bias towards developed and populated areas, we combined the filtered data from our GBIF occurrence download^37^ for each year with the annual data from agricultural agencies in each of the four states, matching the data temporally. Because we used different data sources which were not sampled in the same manner, we looked at occurrences within counties rather than abundance^42^. For the GBIF data, we converted presence point data to county level occurrence. Using R, version 4.2.1^43^, we layered latitude and longitude coordinates from the GBIF observations and trap locations for each year (2017–2021) onto the county geographies from the tigris package^44,45^ to convert our point data into county data.

The state data sets were individually transformed to county presence/absence as follows. For Nebraska counties, we considered the county map data as true presences or absences as they were actively trapping for Japanese beetles in all counties. In Kansas, active monitoring is also occurring, but not in every county as the Japanese beetle has not spread through much of western Kansas. For Kansas counties, it was considered an absence if there were either zero Japanese beetles in their traps or trapping was not occurring. However, in Iowa where the maps are made based solely on reports of Japanese beetle presence, we treated the counties with no Japanese beetle sightings reported as “NA”. For Missouri, the trapping data did not include all counties, so counties with zero Japanese beetles in their traps were treated as true absences while counties without traps set out were left as “NA”.

To create our final data set, we combined the county level GBIF and state datasets described above. The four states (Iowa, Nebraska, Missouri, and Kansas) had a total of 412 counties, and each county was marked with whether Japanese beetle was present in either of the data sources (GBIF or state) or absent from both of them each year for five years (2017–2021). If a county was marked as “NA” in the state dataset and did not have a GBIF sighting, then we could not differentiate true absences from under sampling in that county (i.e., “unknown” data). These counties (12% of the observations) were left as “NA” in the dataset and omitted from the model training data and model testing calculations.

### Generalized additive models

We created generalized additive models (GAMs) to examine the effects of land cover type (corn and soybeans, grassland and pasture, developed areas), climate (average temperature, maximum temperature, minimum temperature, precipitation), state (Nebraska, Iowa, Missouri, and Kansas), and year (2017, 2018, 2019, 2020, 2021) on Japanese beetle presence/absence using the mgcv package^46,47^. The variable of state was included to control for administrative differences within these political boundaries^31^as the status of Japanese beetle infestation, as well as the monitoring of Japanese beetles varies across the four states. We tested for multicollinearity using the variance inflation factor (VIF) function from the package car^48^. The average temperature variable had a very high VIF (> 300), and its effect was similar to the minimum temperature variable. Since minimum temperatures may indicate inhospitable areas due to the lower thermal limits of overwintering Japanese beetle larvae^15,16^we removed average temperature from our models.

We fit generalized additive models using binomial distributions with a logit link function. We performed model selection starting with our full hypothesis model, which included all of our predictor variables: county-level proportion of corn and soybean (ranging from < 0.001 to 0.886), grassland and pasture (ranging from 0.005 to 0.978), and developed areas (ranging from 0.002 to 0.928), maximum temperature (ranging from 11.278 to 22.389), minimum temperature (ranging from − 0.667 to 10.778), precipitation (ranging from 221.7 mm to 1794.7 mm), state (Nebraska, Iowa, Missouri, and Kansas), and year (2017, 2018, 2019, 2020, 2021). We compared our full model to simpler versions, removing variables at each step. We confirmed that all the variables we chose added value over the simpler options using AIC comparisons to find the strongest model^49^. The strongest model was the model with the lowest AIC score, however if models were within 2 delta AIC of one another, the simpler model was chosen.Model validation was performed by using the 2017–2020 data as a training set and holding back the 2021 data as a test set. We compared the 2021 model predictions with the actual 2021 occurrences, and we measured the accuracy of the models using Zero-One scoring^50^.

### Forecasting data

We used the model with the lowest AIC to predict the spread of Japanese beetles in two ways: (1) into the near future (2025 and 2030) under current climate conditions, and (2) using future climate scenarios (RCP 2.6 and RCP 8.5), as described in the Physical Science Basis report of the Intergovernmental Panel on Climate Change^51^. First, we modeled our data forward in time ~ 5 years and ~ 10 years to visualize the spread of Japanese beetles under current climate conditions. To build the predictive dataset, we used 5-year averages (2017–2021) of land cover and climate data to represent the current climate and land cover conditions. We used the most recent 5-year averages versus 30-year climate normal because the biology and spread of Japanese beetle is more likely to be affected by current climate conditions. We changed the *year* variable in our predictive dataset to project the Japanese beetle spread forward in time. For future climate scenarios, we used the year 2030 to account for realistic further spread. We forecasted two different climate scenarios based on a better-case scenario for climate change and a worst-case scenario^51^. For both future climate scenarios, we kept the land cover data as current five year averages (2017–2021). The first scenario is based on RCP 2.6; for this we adjusted our 5-year average dataset with a one-degree Celsius temperature increase and a 10% increase in precipitation^51^. The second scenario is based on RCP 8.5; for this we adjusted our 5-year average dataset with a four-degree Celsius increase in temperature and a 10% decrease in precipitation^51^. Unfortunately, predictive intervals are not available for GAMs, so we were not able to estimate them^46^.

## Results

### Model performance and validation

**Fig. 2:**
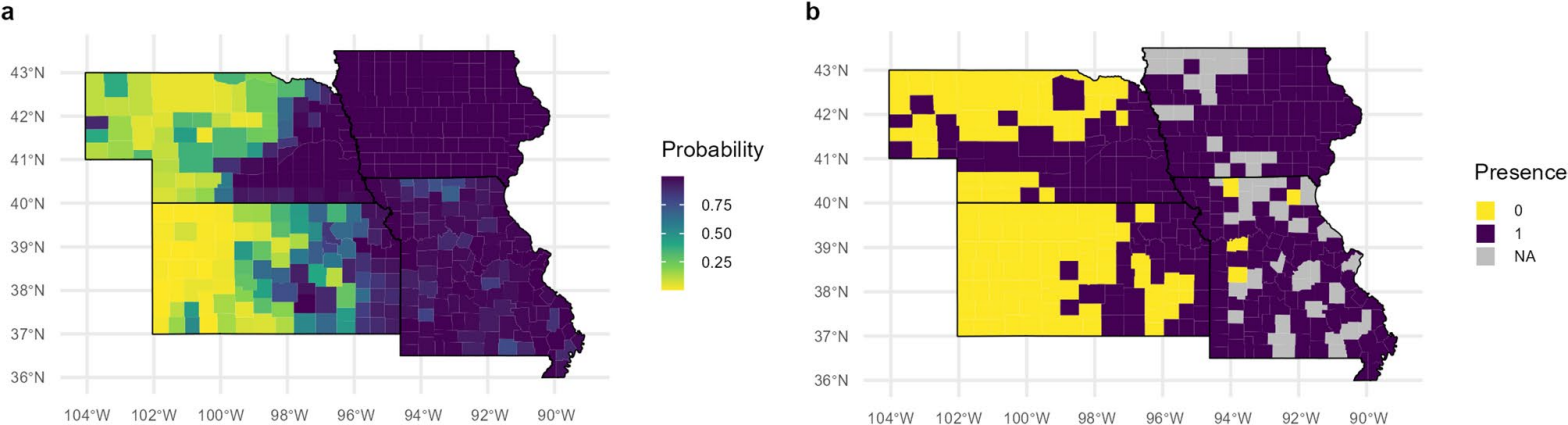
Comparison of the model prediction values for Japanese beetle presence for 2021 (**a**) with the actual presence values in 2021 at the county levels (**b**). For actual presence values, 1 indicates that Japanese beetles were present in the county, 0 indicates that they were absent, and NA indicates that the status was unreported to both data sources

The model with the lowest AIC score included developed areas, corn and soybean, grassland and pasture, precipitation, minimum temperature, maximum temperature, state and year. With Zero-One scoring, our model, trained with 2017–2020 data, had an accurate prediction rate of 90.6% and correctly predicted the status of 326 of the 360 counties in 2021 (Fig. 2). The model was better at predicting true positives (94.9%) than true negatives (80.3%).

### Relationships with predictor variables

**Fig. 3:**
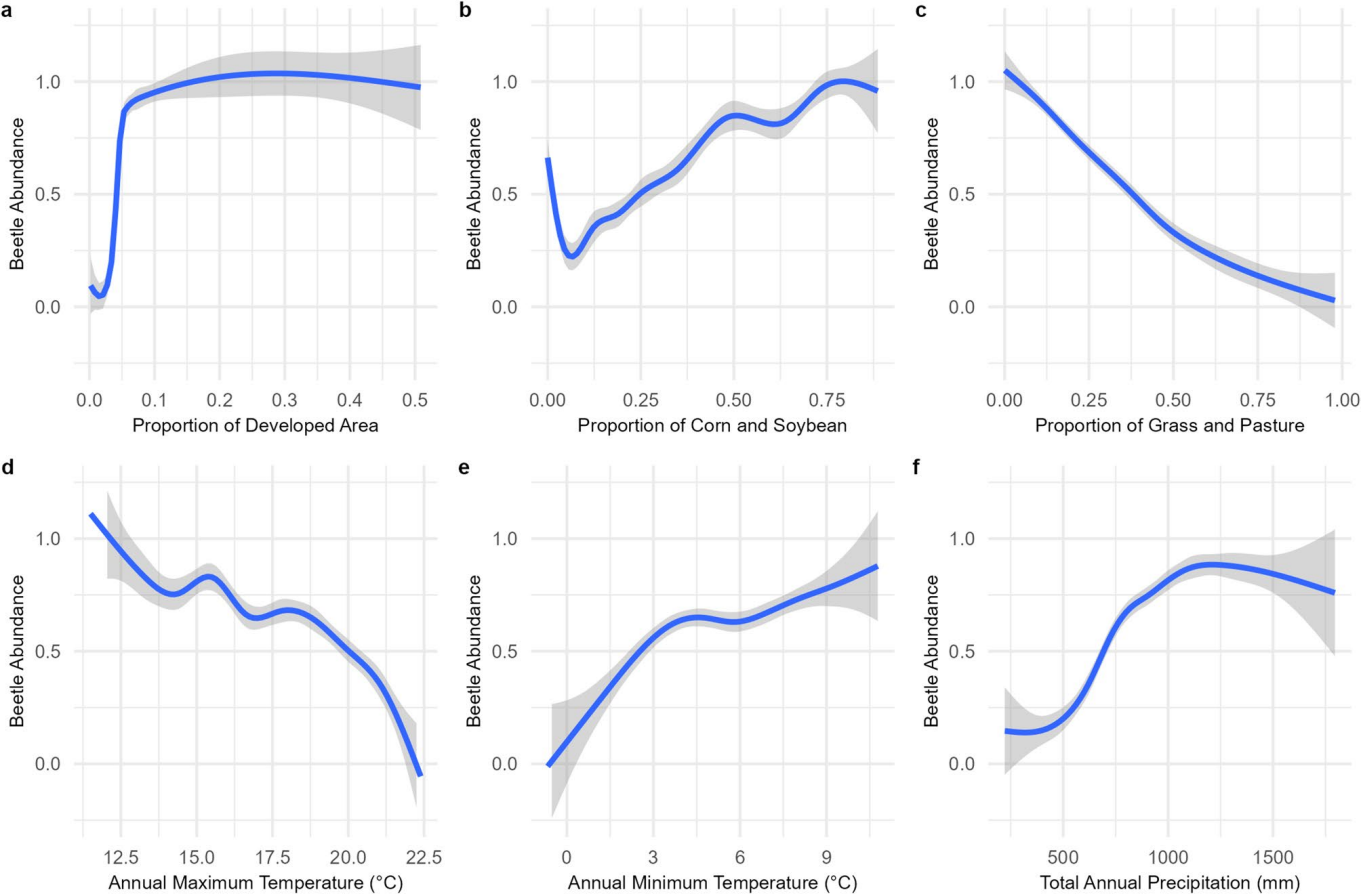
Relationships between each land cover and climate variable and Japanese beetle presence/absence shown with a smooth line. The grey shading represents the 95% confidence intervals. The probability of use varies with proportion of developed areas (**a**), proportion of corn and soybean (**b**), proportion of grassland and pasture (**c**), annual mean maximum temperature in degrees Celsius (**d**), annual mean minimum temperature in degrees Celsius (**e**), and total annual precipitation in mm (**f**)

For land cover variables, there were significant relationships between Japanese beetle presence and developed areas, corn and soybean, and grassland and pasture (Table 1). Developed areas had a very strong positive relationship with Japanese beetle presence, with high probability of presence reached with even a small proportion (0.10) of developed areas (*p* < 0.001, Fig. 3a). Agricultural land planted with corn and soybean had a significant positive relationship with Japanese beetle presence (*p* < 0.05, Fig. 3b). Finally, there was a significant negative relationship between Japanese beetle presence and grassland and pasture (*p* < 0.01, Fig. 3c).

**Table 1:** Model relationships between Japanese beetle occurrences and climate and land cover variables (r^2^ = 0.692).

Predictive Variable	edf	Ref.df	Chi sq.	*P*
Developed Areas	3.704	4.304	62.29	< 0.001
Corn and Soybean	7.195	8.136	19.28	< 0.05
Grassland and Pasture	8.688	8.959	26.67	< 0.01
Maximum Temperature	2.898	3.665	15.20	< 0.01
Minimum Temperature	4.205	5.309	12.78	< 0.05
Precipitation	6.440	7.591	20.52	< 0.05

For climate variables, maximum temperature, minimum temperature and precipitation were also all significant. Maximum temperature had a significant negative relationship with Japanese beetle presence (*p* < 0.01, Fig. 3d). Japanese beetle presence increased steadily as minimum temperature increased (*p* < 0.05, Fig. 3e). Precipitation had a significant positive relationship with Japanese beetles which plateaued around 1000 mm of annual precipitation (*p* < 0.05, Fig. 3f). There were also significant relationships with the state and year variables, indicating the importance of time and space (Table 2).

**Table 2:** Model relationships between Japanese beetle occurrences and state and year (r^2^ = 0.692).

Coefficient	Estimate	Std. Error	z value	Pr(>|z|)
Intercept (Kansas)	1298.000	233.700	−5.555	<0.001
Iowa	63.660	3.961 e^06	0.000	0.999
Missouri	1.062	0.368	2.889	<0.01
Nebraska	3.135	0.572	5.484	<0.001
Year	0.643	0.116	5.556	<0.001

### Model predictions

**Fig. 4:**
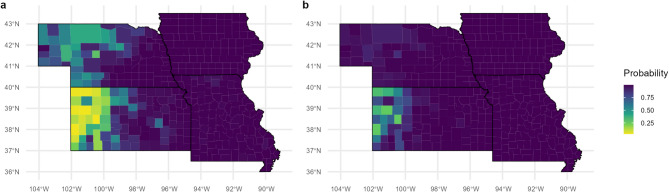
GAM predictions for Japanese beetle suitability per county for 2025 (**a**) and 2030 (**b**) under average climate and land cover conditions for 2017–2021

We modeled the expansion of the Japanese beetle invasion in two ways. First, we used average climate conditions over the last 5 years to predict spread over the next few years (2025 and 2030). Using this approach, we found that Japanese beetles will likely spread out from the current infested counties, beyond the areas predicted from previous models^19,26^ and into the western areas of Nebraska and Kansas (Fig. 4a). Nebraska is predicted to be more suitable than Kansas, with southwestern Kansas having the lowest suitability (0–0.25), even into 2030 (Fig. 4b).

**Fig. 5:**
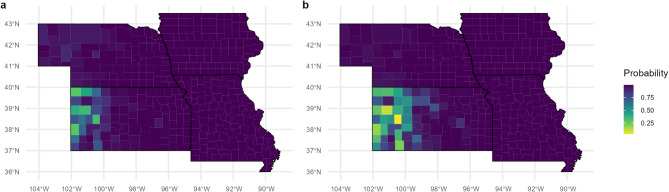
GAM predictions for Japanese beetle suitability per county under future climate scenarios, for the RCP 2.6 scenario with a temperature increase of 1 °C and a 10% increase in precipitation (**a**) and the RCP 8.5 scenario with a temperature increase of 4 °C and a 10% decrease in precipitation (**b**) and average land cover conditions for 2017–2021

Next, we modeled Japanese expansion under future climate scenarios. Our predictions for Japanese beetle presence under an RCP 2.6 climate scenario (a one-degree Celsius increase and ten-percent precipitation increase) suggest that a similar area will be suitable to Japanese beetles as under current climate conditions, but the increase in precipitation in the western region will slightly increase its suitability (Fig. 5a). Our predictions for Japanese beetle presence under an RCP 8.5 climate scenario (a four-degree Celsius increase and ten-percent precipitation decrease) suggest a range compression, mainly in the southwestern area of the region due to the combined hotter and drier climate (Fig. 5b).

## Discussion

The main objectives of this study were to infer regional habitat associations for the Japanese beetle in the American Midwest and Great Plains, and to predict the future spread of the Japanese beetle, specific to this region. We used yearly presence and absence observations of the Japanese beetle over a 5-year period of diffusion at the leading edge to guide our inference and predictions. We identified habitat associations with developed areas, agricultural land planted with corn and soybean, and grassland and pasture, as well as associations with key climatic variables; minimum and maximum temperature and precipitation (Fig. 3). Our model predictions indicate a higher probability of Japanese beetle infestations in this area than previously estimated^19,25^with only slight changes under future climate scenarios. This difference could be due to differences in the temporal and spatial extents of our study and the use of county and extension data to supplement community science data. We discuss these issues in greater detail below.

We found that all land cover variables, the percentage of developed areas, corn and soybean, and grassland and pasture, had significant relationships with Japanese beetle presence. As expected, developed areas had strong significant positive relationship with Japanese beetle presence. Counties with 10% or more developed area are highly likely to have Japanese beetles (Fig. 3a). Sharp increases in the probability of Japanese beetle infestation, even at low levels of developed areas, could be due to the fact that these developed areas provide important habitat for the development of Japanese beetle larvae, such as turfgrass^14,15^. Additionally, Japanese beetles are known to hitchhike into new regions on planes, trains, and automobiles in a form of unintentional human-mediated transport^52^. Higher proportions of developed areas also indicate more populated counties with higher possibilities for human mediated dispersal.

The amount of corn and soybean also had the expected significant positive relationship with beetle presence (Fig. 3b). This supports our hypothesis based on Japanese beetle biology, as adults feed on field crops such as soybean and corn, and they have also been found to oviposit in field crops, with a preference for soybean and no-till fields^16^. Japanese beetles use resources from both developed areas and agricultural fields throughout their life cycle. Previous studies have found that interactions between agriculture and other areas are especially important for Japanese beetles. Della Roca^53^ found that Japanese beetles were more likely to occupy areas with not only a large amount of agriculture, but which also provided high habitat diversity. Borner et al.^26^ also cited the availability of multiple food sources as an explanation for why broad-leaved forests in proximity to open space were the most relevant land-use variable. A mixture of agricultural and developed land cover types could benefit Japanese beetles because they use resources from both at different points in their life history. Although as adults Japanese beetles are considered a generalist species with over 300 host plants^14–16^females show a preference for specific oviposition conditions, such as short grass cover^15,54^ and moist soils^15,55^and larval diet consists mainly of grass roots^14–16,56^. Agricultural areas near developed areas with turfgrass and other larval habitat are likely the most at risk for Japanese beetle infestation.

However, we found a negative relationship between the probability of Japanese beetle presence and the amount of grassland and pasture in the county (Fig. 3c). This was unexpected as grass roots are the primary diet of Japanese beetle larvae^14–16^ and it was predicted that managed pastures would allow Japanese beetles to thrive in areas of the central United States where it would otherwise be too dry^19^. Japanese beetles might consider grasslands of the Midwest as an undesirable natural area, despite prior research that females deposit their eggs in grass and pasture^14,16^. Although this is the opposite of what we would expect based on established Japanese beetle populations in the eastern United States^14^a recent study in northern Italy also found that the probability of Japanese beetle presence decreased with higher percentages of pastures^53^. This could be due to the variation in grassland composition across these different regions. For example, there are 65 distinct types of grasslands across the world all classified as “grassland”^57^. Grasslands have distinctive characteristics, such as grass species, soil moisture, height, and management practices. Tallgrass prairies of the Midwest and Great Plains have characteristically tall, dense, stands of grass with deep root systems^58^. These root systems may be problematic for grubs that feed on grass roots and move up and down in the soil profile from fall to spring. It could also be due, in part, to an increase in predators of the Japanese beetle, such as grassland birds^59^.

Unfortunately, recent demand for sustainable fuels have increased cropland for corn and soybean in the Midwest^60^ with the majority of the land coming from converting grasslands which were previously Conservation Reserve Program (CRP) land and pasture^61,62^. A study looking urbanization across the United States found that impervious surface area also increased by around 11% over a decade, and that the impervious surface area per person was highest in the Midwest^63^. While we did not model how land cover change scenarios would affect the distribution of the Japanese beetles, the future expansion of cropland or urbanization at the expense of grassland could expedite the expansion of the Japanese beetle’s range. Based on the negative association of grasslands and Japanese beetle occurrence in our model, the conservation of native grasslands could be helpful in slowing the spread of Japanese beetles.

Temperature and precipitation are known to affect Japanese beetle, and in our model, maximum temperature, minimum temperature, and precipitation had significant relationships with Japanese beetle presence (Table 1). From our analysis, we can see that maximum temperature has a negative relationship with beetle presence (Fig. 3d). While warmer temperatures are conducive to Japanese beetle population growth, higher maximum temperatures could have a negative effect on Japanese beetles, as beetle eggs will not hatch above 34 °C^14^ and decreased numbers of Japanese beetles in Arkansas were attributed to hotter summers^64^. Niziolek et al.^65^ found that Japanese beetles consumed more leaf matter in higher temperatures, but those that fed at elevated temperatures also exhibited increased mortality, especially above 37 °C. Our results also indicate a significant positive relationship with minimum temperature (Fig. 3e). Minimum temperature corresponds to the lower thermal limit at which the larvae can survive, and areas with extremely cold winters are inhospitable to Japanese beetles as their supercooling point is − 7 °C^15^. Therefore, higher minimum temperatures are likely to lead to greater survival of Japanese beetle larvae. Our model shows a generally positive relationship between precipitation and the probability of beetle presence, but the positive relationship plateaus at around 1000 mm (Fig. 3f). While lower precipitation can deter Japanese beetle presence, our models show that under the current climate conditions, even most drier portions of western Kansas are likely to have Japanese beetles with the passing of time. This may be because although Japanese beetles are known to oviposit in soils with a moderate to high moisture content, it is only the eggs and first instars which are sensitive to desiccation^14,66^. Therefore, areas with higher moisture such as lawns and irrigated crop fields could provide adequate habitat even during times of drought^15,19^.

For future predictions, our results show that Japanese beetles will quickly spread across the American Midwest and Great Plains. Although previous models based on global occurrences of Japanese beetles have shown lower suitability for a large portion of the western area of our study region^19,25,26^ our model suggests that this area is more suitable than previously thought. Given the conflicting relationships with minimum and maximum temperature, we would expect global temperature increases to expedite the spread of Japanese beetles only up to a certain point. The increase of temperature minimums would decrease losses of Japanese beetle eggs or larvae in the cooler months. However, an increase in maximum temperature could cause mortality in the summer months. Changes in precipitation can also affect Japanese beetle, with increased precipitation favoring quicker expansion and greater abundance. Low precipitation has long been predicted as a barrier to Japanese beetle infestation west of the 100th meridian^14^ and a decrease in precipitation could slow the spread of Japanese beetles to that area. Indeed, when we forecasted for the presence of Japanese beetles under two climate scenarios (RCP 2.6 and RCP 8.5), our model concurred with these expectations. In an RCP 2.6 scenario, with an increase in temperature as well as precipitation, the probability for Japanese beetle presence increased in the western areas of this region (Fig. 5a). However, with the RCP 8.5 scenario, which included an even higher temperature increase but a precipitation decrease, our models suggest that the southwestern portion will become less hospitable to Japanese beetles (Fig. 5b). Our climate predictions are similar to predictions by Kistner-Thomas (2019) which also show a northeastern range-shift for suitability under the RCP8.5 scenario and diverge from predictions by Della Roca and Milanesi (2022) which show high suitability for this entire region under future climate scenarios. Our model’s future climate predictions are in-line with observed effects of increased temperature on Japanese beetle mortality in experimental settings^65^ and abundance in field settings^64^.

Differences between our model predictions and previously published predictions^19,25^ could be due in part to the rural composition in these areas, as we used state agency and University extension data to supplement the GBIF data which increased Japanese beetles occurrence within the counties by more than two-fold. Without this addition of these data from rural and agricultural areas, relationships with land cover and climate variables could be missed, especially along this invasion front. The differences between our models and previous global models could also be due to differences in the temporal and spatial extents of the models. Habitat associations can vary by region^32,67^ and these associations can be obscured if models are created at a much broader spatial scale such as global models^30^. On the other hand, if a spatial extent is too small, habitat associations can be clouded by differences in sampling and management efforts across political boundaries. In our model, the significant effect of state indicates both an effect of location, as Japanese beetles are spreading east to west, and an effect of varying degrees of effort to locate and manage Japanese beetles between the states.

Additionally, time is particularly important for species distribution mapping along an invasion front. Without the temporal variable, the effect of diffusion is lost, which is particularly important in areas where invasive species are expanding their range and there is suitable area which has not yet been utilized^33^. Our predictions show that the western spread of Japanese beetles farther into Nebraska and Kansas is more a matter of time than habitat and climate associations. By capturing the expansion of Japanese beetles into new counties over the 5-year period of our study, we were able to model to the near future with current climate and land cover conditions, and without any climate changes, we see that they will likely be present in every county of the region. Our current data and modeling approach enables statistical inference and prediction/forecast at the county level, which is useful for larger scale, regional management of invasive species. Future work for Japanese beetle distribution in this region could focus on abundance data at the point level, which would be more relevant if done in specific land cover types. Such future crop-specific work could be helpful to inform stakeholders of their risk at a more refined level.

## Conclusion

In summary, we found that developed areas had a very strong positive relationship with Japanese beetle presence, even at low proportions within the county. Corn and soybean also had a strong positive association with Japanese beetle presence. However, grassland and pasture had an unexpected negative relationship with Japanese beetle presence. This finding underscores the importance of modeling habitat associations at a regional scale, as ecosystems such as grasslands can have regional differences such as species composition, soil morphology, root architecture, and disturbance regimes. This study also provides an example for mitigating bias in community science data by integrating systematic agricultural observations to more accurately evaluate the spread of invasive pests within agroecosystems. Future studies on invasive pests would benefit from accounting for temporal dynamics and enriching citizen science data with agricultural or rural datasets when tracking and predicting invasions. Models incorporating time as a factor at a regional scale, such as ours, can also help identify areas at higher risk of early infestation, enabling more strategic monitoring and resource allocation.

## Supplementary Information

Below is the link to the electronic supplementary material.

Supplementary Material 1

## Data Availability

Data is provided within the manuscript or supplementary information files.

## Declarations

### AI use

Authors have not used AI-assisted technologies in creating this article.

### Data statement

Data is provided within the manuscript or supplementary information files.
